# A Flexible Multifunctional PAN Piezoelectric Fiber with Hydrophobicity, Energy Storage, and Fluorescence

**DOI:** 10.3390/polym14214573

**Published:** 2022-10-28

**Authors:** Qisong Shi, Rui Xue, Yan Huang, Shifeng He, Yibo Wu, Yongri Liang

**Affiliations:** 1 College of New Materials and Chemical Engineering, Beijing Institute of Petrochemical Technology, Beijing 102617, China; 2 State Key Lab of Metastable Materials Science and Technology, School of Materials Science and Engineering, Yanshan University, Qinhuangdao 066004, China

**Keywords:** piezoelectricity, polyacrylonitrile, ionic liquid, energy storage, sensors

## Abstract

Lightweight, flexible, and hydrophobic multifunctional piezoelectric sensors have increasingly important research value in contemporary society. They can generate electrical signals under the action of pressure and can be applied in various complex scenarios. In this study, we prepared a polyacrylonitrile (PAN) composite fiber doped with imidazolium type ionic liquids (ILs) and europium nitrate hexahydrate (Eu (NO_3_)_3_·6H_2_O) by a facile method. The results show that the PAN composite fibers had excellent mechanical properties (the elongation at break was 114% and the elastic modulus was 2.98 MPa), hydrophobic self-cleaning ability (water contact angle reached 127.99°), and can also emit light under UV light irradiation red fluorescence. In addition, thanks to the induction of the piezoelectric phase of PAN by the dual fillers, the composite fibers exhibited efficient energy storage capacity and excellent sensitivity. The energy density of PAN@Eu-6ILs reached a maximum of 44.02 mJ/cm^3^ and had an energy storage efficiency of 80%. More importantly, under low pressure detection, the sensitivity of the composite fiber was 0.69 kPa^−1^. The research results show that this PAN composite fiber has the potential to act as wearable piezoelectric devices, energy storage devices, and other electronic devices.

## 1. Introduction

Wearable electronic products have developed rapidly in recent decades [1], and they are mostly used in the medical and health field to monitor human body signals or as self-powered generators as new energy devices in the future [2,3]. Compared with traditional electrical materials, which are generally rigid and unsuitable for flexible devices [4], nanofiber-based electronic products can achieve light weight and stretchable flexibility, which ensures structural integrity under a certain tensile deformation [5]. Flexible and wearable pressure sensors can respond to external pressure stimuli and convert them into electrical signals [6], and the capacitive piezoelectric sensor, as the one of the most dazzling of the new generations of piezoelectric sensors, has high-quality features such as simple and efficient device structure, high sensitivity, low loss, and fast response speed [7,8]. It can continuously accumulate the internal charge of the material under pressure to achieve self-powering functionality [9]. The distance between electrodes and the high dielectric constant of the dielectric layer has a crucial impact on capacitive piezoelectric sensors [10]. At the same time, the products prepared by electrospinning technology are often disturbed by the influence of moisture in the air and sweat produced by the human body [11]. Therefore, the development of a capacitive flexible piezoelectric sensor with high dielectric constant, high sensitivity, and hydrophobicity has become a research hotspot. To meet the requirements of energy and environmental sustainability [12], there is an urgent need to develop multipurpose sensors with energy storage or self-powering. There are recent research reports. For example, Khalifa [13] et al., reported a poly(vinylidene fluoride) PVDF/mica nanosheet composite (PMNCs) prepared by solution casting method with a high sensitivity of 3.2 N^−1^ and capable of generating a maximum output voltage of 32 V under a pressure of 5 N, which was used in wearable energy storage and piezoelectric sensors. Wang [14] et al. reported a hybrid nanogenerator with self-powered and simultaneous energy storage through a power management circuit. This hybrid nanogenerator had a maximum output power of 1.7 mW when the load resistance was 10 MΩ, and the energy storage efficiency was as high as 112%.

Polyacrylonitrile (PAN) is a kind of piezoelectric polymer. PAN nanofibers prepared by electrospinning technology have the advantages of appropriate piezoelectric properties, good mechanical properties, and high piezoelectric sensitivity, which makes it widely used in the field of flexible piezoelectric sensors [15]. PAN is an amorphous vinyl-type polymer containing a cyano group (-CN) in each repeating unit [16]. PAN has two typical conformations, planar zigzag conformation and 3^1^ helical conformation [17], and the planar zigzag conformation has a total transformation (TTTT) structure [18]. Compared with the most popular piezoelectric polymer polyvinylidene fluoride (PVDF), it has a stronger dipole moment of 3.5 Debye than the β phase (2.1 Debye) of PVDF [19]. PAN also has smaller dielectric loss and good thermal stability [20]. Therefore, PAN is expected to be a better piezoelectric material than PVDF. Ionic liquid (ILs) is a conductive material composed entirely of ions [21] that has high ionic conductivity, electrochemical stability, and other properties [22]. It was reported that good dielectric properties and improved mechanical properties were obtained by incorporating ionic liquids into polymer systems [23]. In addition, mono-, binary, or ternary sensor systems combined with ionic liquids have also been widely developed and utilized. Jiang [24] et al. reported a strain sensor of TPU/ILs. The sensor had a maximum tensile limit of 400%, a high sensitivity of 1.28 and a fast response of 67 ms under high strain conditions. Zhang [25] et al. prepared a PVDF dielectric composite material doped with ILs and graphene. When the concentrations of graphene and ILs both reached 2 wt%, the composite material achieved the best dielectric properties of about 7, which was nearly six times that of PVDF, and the dielectric loss was lower than 0.2. Yuan [26] et al. designed a ternary composite of RGO@ILs/PBO. The mechanical properties and dielectric properties of the composite material were greatly enhanced. Its dielectric constant at 10^3^ Hz and 200 °C was 35.51, and the dielectric loss was 0.09. Meanwhile, its application in energy storage had an energy density as high as 2.38 J/cm^3^ and a breakdown strength of 122.96 kV/mm. In addition, fluorescent nanofibers were prepared by doped fluorescent fillers with piezoelectric polymers, which could be applied to self-powered optoelectronic devices. Fu [27] et al. prepared a polyvinylidene fluoride hexafluoropropylene (PVDF-HFP) composite fibers with addition of Eu (TTA)_3_(TPPO)_2_ and BaTiO_3_ by electrospinning technology. The results showed that it can display stable red fluorescence and had a piezoelectric sensitivity of 0.49 kPa^−1^ and an energy storage density of 30.45 mJ/cm^3^.

PAN composite fibers doped with ILs and (Eu (NO_3_)_3_·6H_2_O) were prepared, and a flexible multifunctional PAN piezoelectric fiber with hydrophobicity, fluorescence, and energy storage was obtained through the synergistic effect of the dual fillers. It can be used in fields such as flexible piezoelectric sensors and energy storage devices. The results show that the content of the planar zigzag phase in the dual-filler PAN@Eu-ILs composite fiber was as high as 94.74%, which demonstrated its high piezoelectric phase content. At the same time, the small interplanar spacing was also proof of the high piezoelectric phase content. The double-filled PAN composite fibers also had a good energy storage density, reaching an energy storage density of 44.02 mJ/cm^3^ under the action of an electric field of 420 kV/cm^3^, which was 1.64 times of the energy storage density of pure PAN (26.84 mJ/cm^3^). More importantly, the dual-filler PAN composite fibers also had a larger tensile deformation range, softer properties, and can exhibit red fluorescence. In addition, the water contact angle of the single-component PAN-ILs composite fiber reached 127.99°, and its excellent hydrophobic properties and self-cleaning ability can avoid the influence of environmental humidity on the sensor. Finally, the PAN composite fiber has a high sensitivity up to 0.69 kPa^−1^ in the pressure range of <1 kPa, and it has a high sensitivity for low pressure detection.

## 2. Materials and Methods

### 2.1. Materials

The chemicals used in this study include polyacrylonitrile (PAN), MW = 85,000, Shanghai MACKLIN Company, Shanghai, China; 1-allyl-3-butylimidazolium tetrafluoroborate [AMIm][BF_4_] (ILs), Lanzhou Institute of Chemical Physics, Chinese Academy of Sciences, Lanzhou, China; europium nitrate hexahydrate (Eu (NO_3_)_3_·6H_2_O), Beijing Warwick Chemical Co., Ltd., Beijing, China; N, N dimethylformamide (DMF), Beijing Chemical Plant, Beijing, China. All samples were used directly.

### 2.2. Preparation of PAN@Eu-ILs Multifunctional Composite Fibers

**Figure 1:**
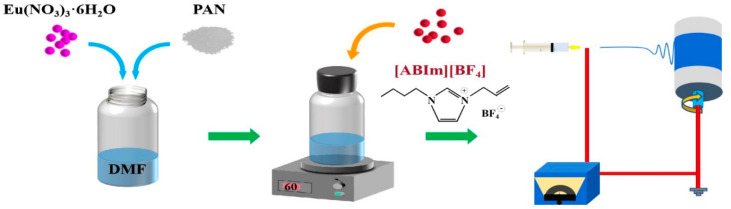
Experimental flow chart.

A total of 1.4 g of PAN was added to 10 mL of DMF solution. The mass fraction of PAN in the solution was 14% (*w*/*v*). It was heated and stirred at 60 °C until the PAN was completely dissolved and the solution was pale yellow. Then, 0.14 g of Eu (NO_3_)_3_·6H_2_O was added to the solution, and the solution was heated and stirred until the Eu (NO_3_)_3_·6H_2_O was completely dissolved. Finally, ILs were added to the solution, where IL levels were 0%, 3%, 6%, and 9% of the mass fraction of PAN, which was named PAN@Eu-XILs (X was the mass fraction of ILs in PAN). The solution was heated and stirred for electrospinning. A 5 mL syringe was used to extract an appropriate amount of spinning solution. The process parameters were as follows. A 20 G metal needle was used on the syringe, and the injection speed was 0.2 mm/min. The spinning distance was 15 cm, the applied spinning voltage was a positive voltage of 16 kV and a negative voltage of 2 kV, and the rotational speed of the drum was 120 r/min. The ambient humidity was 45–50%. A schematic diagram of the experimental preparation is shown in Figure 1.

### 2.3. Characterization

The SSX-550 model SEM (Shimadzu Co., Kyoto, Japan) was used to study the surface morphology and orientation of nanofibers. The diameters of the nanofibers in the SEM images were statistically calculated by Image J software. EDS images were tested by a Sigma 300 model Oxford Energy Spectrometer (Carl Zeiss Co., Jena, Germany). All samples were treated with gold spray. Nicolet-380 Fourier Transform infrared spectrometer (Thermoelectric Co., West Chester, PA, USA) was used to determine the chemical structure of nanofibers, and the vibration bands areas were calculated by OMINC software analysis. The FS5 spectrofluorometer (Edinburgh Instruments, SCT, UK) was used to analyze the fluorescence properties of composite fibers. The crystal structure of composite fibers was studied by XRD-7000 X-ray diffractometer (Shimadzu Co., Kyoto, Japan). The SL200KS optical contact Angle and surface tensionmeter (Solon Information Technology Co., Ltd., Shanghai, China) was used to measure the contact Angle of water. The DSC and TG of the composite fibers were measured using a synchronous thermal analyzer from setline, the heating rate was 10 °C/min, and the temperature curve was 35–800 °C. The ferroelectric energy storage performance of composite fiber was studied by TF analyzer 2000 (aixACCT Co., Eschweiler, Germany). The conductive tape was tightly attached to the upper and lower surfaces of the sample. The ferroelectric test was carried out at room temperature with a test frequency of 10 Hz and a test voltage range of 0.1–10 kV. GP6220 High temperature tensile testing machine (Gaopin Testing Instruments Co., LTD., Suzhou, China) was used to test the mechanical properties of composite fibers. The sample was cut into a size of 40 mm × 10 mm, the test temperature was 25 °C, and the test speed was 10 mm/min. The BDS40 broadband dielectric impedance spectrometer (NOVOCONTROL Co., Montabaur, Germany) was used to measure permittivity and dielectric loss. The test range was 10^1^ to 10^6^ Hz. The TH2828 LCR digital bridge from Changzhou Tonghui Electronics Co., Ltd. Changzhou, China was used to test the sensitivity of composite fibers. The sample was cut into a size of 20 mm × 20 mm, and the conductive tape was tightly attached to the upper and lower surfaces of the sample. Under the test conditions of 1.5 V and 100 Hz frequency, the composite fiber was subjected to a pressure test and the change in the capacitance value was recorded.

## 3. Results

### 3.1. SEM

**Figure 2:**
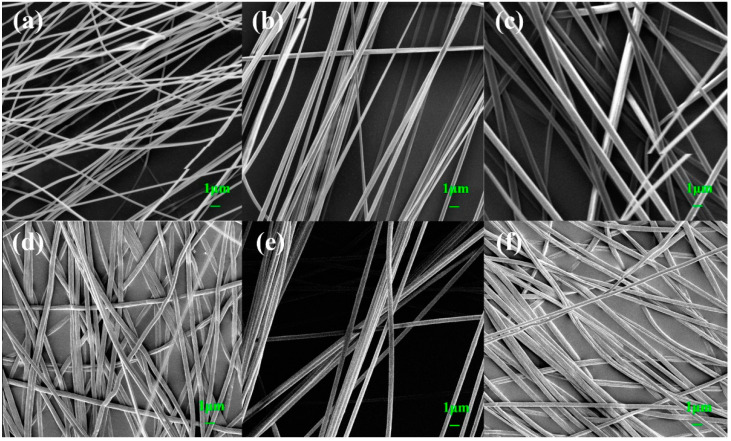
SEM of PAN@Eu/ILs composite fibers: (**a**) pure PAN; (**b**) PAN-6ILs; (**c**) PAN@Eu; (**d**) PAN@Eu-3ILs; (**e**) PAN@Eu-6ILs; (**f**) PAN@Eu-9ILs.

**Figure 3:**
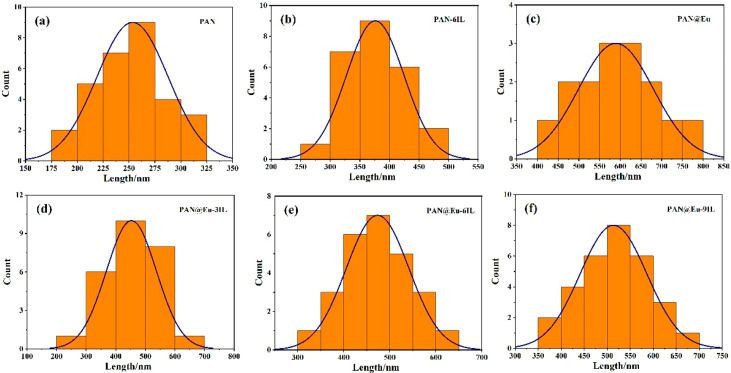
Diameter distribution of PAN@Eu/ILs composite fibers: (**a**) pure PAN; (**b**) PAN-6ILs; (**c**) PAN@Eu; (**d**) PAN@Eu-3ILs; (**e**) PAN@Eu-6ILs; (**f**) PAN@Eu-9ILs.

**Figure 4:**
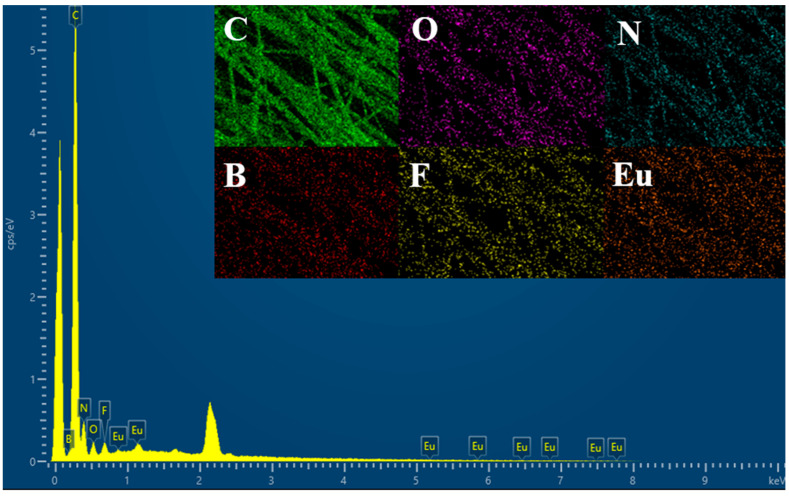
EDS images of PAN@Eu-6ILs composite fibers. The inset shows the EDS distribution image of each element in the PAN@Eu-6ILs composite fiber: C in PAN@Eu-6ILs, O in PAN@Eu-6ILs, N in PAN@Eu-6ILs, B in PAN@Eu-6ILs, F in PAN@Eu-6ILs, Eu in PAN@Eu-6ILs.

It can be seen from Figure 2 that the fiber diameter was uniform, and the surface was smooth, indicating that the electrospinning process was not affected by the external environment and the spinning process was relatively stable. The composite fiber with a single addition of IL displayed a certain orientation. This may be due to the high charge density of the solution, which was formed by the increase in the ionic conductivity of the solution by ILs [28]. Under the action of the high electric field force, the extensional flow of the solution under the action of the electrostatic force was enhanced, which was why the composite fiber exhibited a certain orientation [29]. However, after the addition of Eu (NO_3_)_3_·6H_2_O and ILs, the strong interaction between them had a certain effect on the orientation of the composite fibers, and the orientation was not as obvious as that of the PAN-IL composite fibers. As the IL concentration increased, the electrostatic force stretching increased, the dipoles became more polarized, and more PAN piezoelectric phases (planar zigzag conformation) were induced to form [15]. As shown in Figure 3, the bar graph represented the fiber diameter distribution, and the peak of the curve represented the average diameter. The average diameters of PAN@Eu-ILs composite fibers were as follows: 253 nm for PAN, 587 nm for PAN@Eu, 375 nm for PAN@ILs, 425 nm for PAN@Eu-3ILs, 475 nm for PAN@Eu-6ILs, and PAN@Eu-9ILs was 515 nm. The average diameter of the composite fibers added with Eu (NO_3_)_3_·6H_2_O became obviously thicker, while that with the addition of ILs decreased slightly. First, after adding Eu (NO_3_)_3_·6H_2_O, the concentration of the solution increased. The higher solution concentration caused the thickest of the fiber diameter of the PAN@Eu. Meanwhile, due to the ionic conductivity of ILs, the spinning solution was stretched by stronger electrostatic force, and the diameter of the composite fibers decreased slightly. The EDS images in Figure 4 show that the composite fibers contain C, O, N, F, B, and Eu elements. It was confirmed that ILs and Eu (NO_3_)_3_·6H_2_O were successfully added and uniformly distributed.

### 3.2. FT-IR

**Figure 5:**
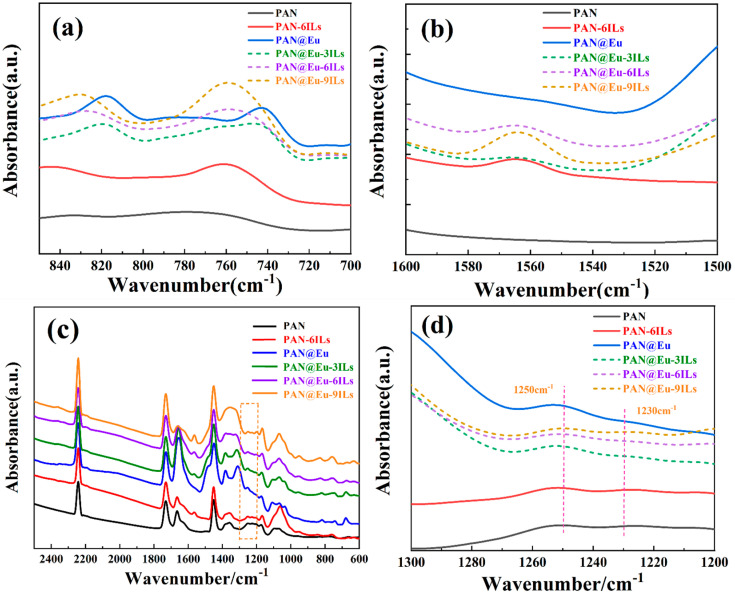
FT-IR spectra of PAN@Eu-ILs composite fibers in the frequency range of (**a**) 850 cm^−1^−700 cm^−1^; (**b**) 1600 cm^−1^−1500 cm^−1^; (**c**) 2500 cm^−1^–600 cm^−1^; (**d**) 1300 cm^−1^–1200 cm^−1^.

Figure 5a–d shows the FTIR spectra of the PAN@Eu-ILs composite fibers. Both PAN nanofibers and PAN composite fibers had similar infrared spectral curves. The characteristic peaks corresponding to PAN were as follows: The vibration bands at 2245 cm^−1^ was attributed to the stretching vibration of the -C≡N bond [30]. The vibrational peaks at 1450, 1360, and 1070 cm^−1^ were formed by the bending vibrations of the -CH_2_-, -CH, and the -CN bonds, respectively [31]. Figure 5a shows that, compared with PAN, the characteristic peaks at 817 cm^−1^ and 741 cm^−1^ of PAN@Eu correspond to the vibrations of (C-N-C(O)) and (O-C-N) in the Eu^3+^-PAN structure, respectively [32]. However, after the PAN@Eu composite fiber was doped with ILs, the vibration bands position shifted toward the lower wavenumber side. The vibration bands positions shifted more with the increase in IL concentration, which reflected the strong interaction between Eu (NO_3_)_3_·6H_2_O and IL. From Figure 5b, we can see that a strong vibration band appeared at 1562cm^−1^, which was attributed to the stretching vibration of the imidazole ring of ILs [33]. With the increase in IL concentration, the vibration bands intensity gradually increased. Figure 5a,b confirms the successful doping of Eu (NO_3_)_3_·6H_2_O and IL on PAN composite fibers. In order to further explore the effect of fluorescent fillers and ILs on the structure of PAN, we analyzed and calculated the two conformations of PAN composite fibers. The vibrational bands of 1250 cm^−1^ and 1230 cm^−1^ correspond to the planar zigzag and 3^1^ helical conformation of PAN, respectively [34]. The planar zigzag conformation was more favorable for the generation of piezoelectric phase. The area ratio of the two peaks can be calculated by Equation (1) [30], thereby obtaining the changes of the two conformations in different PAN composite fibers, and further deducing the effect on the piezoelectric properties.

(1) $$\Phi =\frac{S_{1250}}{S_{1250}+S_{1230}}$$

where *S*_1230_ and *S*_1250_ represent the peak areas at 1230 cm^−1^ and 1250 cm^−1^, respectively. It can be seen from Figure 5d that after adding Eu (NO_3_)_3_·6H_2_O and ILs, the intensity of the vibration bands at 1250 cm^−1^ was improved, indicating that the 3^1^ helical conformation gradually changed to a planar zigzag conformation. In general, the higher the content of the planar zigzag conformation, the higher piezoelectric properties of PAN nanofibers [35]. The Φ of PAN@Eu reached 90.04%, the Φ of PAN-6ILs reached 92.01%, and the Φ of PAN@Eu-6ILs reached 94.74%. Compared with the PAN nanofibers (Φ reached 85.06%), the composite fillers with ILs and Eu (NO_3_)_3_·6H_2_O simultaneously played a synergistic effect on the generation of the piezoelectric phase of PAN, which had the best piezoelectric performance. Therefore, when the amount of addition was appropriate, the best piezoelectric material can be obtained.

### 3.3. XRD

**Figure 6:**
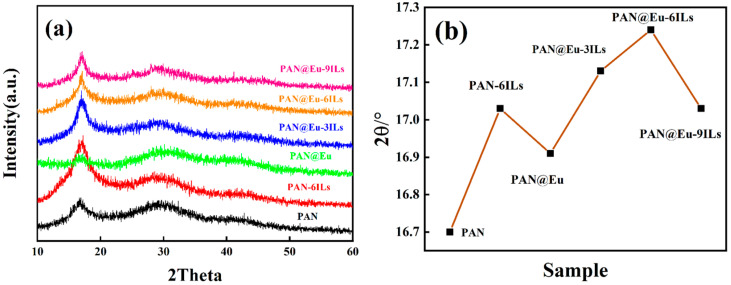
(**a**) XRD patterns of PAN@Eu-ILs composite fibers; (**b**) the position of the (100) crystal plane diffraction peak of PAN@Eu-ILs composite fibers.

Figure 6a shows the XRD patterns of the PAN@Eu-ILs composite fibers. For pure PAN, the main characteristic peak at 2θ = 16.7° corresponded to the (100) facet peak of PAN [36], and the broad peak at approximately 2θ = 29° was assigned to the rotational disorder in the chain packing [37]. It was obvious that the crystal plane diffraction peak position of (100) was shifted to the right after adding ILs and Eu (NO_3_)_3_·6H_2_O. In general, the right shift of the diffraction peak indicated that the addition of fillers promoted the transition from the 3^1^ helical conformation to the planar zigzag conformation [38], which was beneficial to the improvement of the piezoelectric properties of the composite fibers. Among them, the (100) crystal plane diffraction peak of the PAN@Eu-6ILs composite fiber shifted to the right the most, which indicated that the plane zigzag conformation content was the most. The results corresponded to the FT-IR, and further verified the synergistic effect of composite fillers helped PAN composite fibers to achieve the best piezoelectric properties. The position statistics of the diffraction peaks of the (100) crystal plane is shown in Figure 6b. The (100) crystal plane positions of PAN@Eu and PAN-6ILs in the single-filler system were 16.91° and 17.02°, respectively, and the diffraction peak intensity of PAN@Eu was weak. In the dual-filler system, when the ILs contents were 3%, 6%, and 9%, the positions of the (100) crystal plane corresponded to 17.13°, 17.24° and 17.02°, respectively. The interplanar spacing (D) can be calculated by the Bragg Equation (2) [39]:

(2) 2dsinθ=nλ

where d is the interplanar spacing of the corresponding crystal plane, and θ and λ are the diffraction angle and X-ray wavelength, respectively. The calculated results show that the interplanar spacings of PAN, PAN@Eu, and PAN-6ILs were 0.532 nm, 0.528 nm, and 0.524 nm, respectively, while in the dual filler system, the interplanar spacings of PAN@Eu-3ILs, PAN@Eu-6ILs and PAN@Eu-9ILs were 0.520 nm, 0.518 nm, and 0.524 nm, respectively. It was evident that the synergistic effect of the dual fillers further reduced the interplanar spacing. According to previous reports, the planar zigzag conformation has a smaller interplanar spacing [40]. This further confirmed the generation of the PAN piezoelectric phase.

### 3.4. DSC and TG

**Figure 7:**
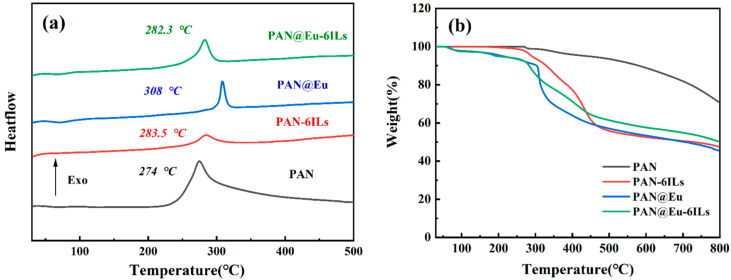
(**a**) DSC spectrum of PAN composite fibers; (**b**) TG curve of PAN composite fibers.

Figure 7 is the DSC and TG curves of PAN composite fibers. As shown in Figure 7a, the PAN composite fiber had a characteristic exothermic peak temperature (T_p_) at 274 °C, corresponding to the cyclization reaction of PAN. The peak temperatures of PAN-6ILs, PAN@Eu, and PAN@Eu-6ILs composite fibers were 283.5 °C, 308 °C, and 282.3 °C, respectively. Because of ILs and Eu (NO_3_)_3_·6H_2_O, the activation energy required for the cyclization of PAN composite fibers increased, and the T_p_ of the composite fibers increased. This indicated that the PAN composite fibers obtained a uniform and stable structure with improved thermal stability [41]. Compared with the single-filler PAN composite fibers, the T_p_ of the dual-filler PAN@Eu-6ILs decreased, which may be the result of the strong interaction between ILs and Eu (NO_3_)_3_·6H_2_O. In addition, the cyclization reaction of different intermolecular cyano groups resulted in a slight elevation of the baseline of PAN composite fibers [42]. 

As shown in Figure 7b, PAN and PAN-6ILs began to lose a significant amount of weight at around 280 °C, which was mainly due to the decomposition of ILs and polymer chains. With the addition of fluorescent fillers, the PAN composite fibers began to dissociate rapidly at 308 °C, the thermal decomposition temperature shifted to high temperature, and the thermal stability was improved. However, the residual weight of PAN at 800 °C s was 71%, which was much higher than that of the PAN composite fibers with Eu (NO_3_)_3_·6H_2_O and ILs added. For example, the residual weight of PAN@Eu-6ILs at 800 °C was only 51%. Although the residual weight decreased, the piezoelectric and ferroelectric energy storage properties in the PAN composite fibers were improved, which was beneficial to its better application.

### 3.5. Dielectric Properties

**Figure 8:**
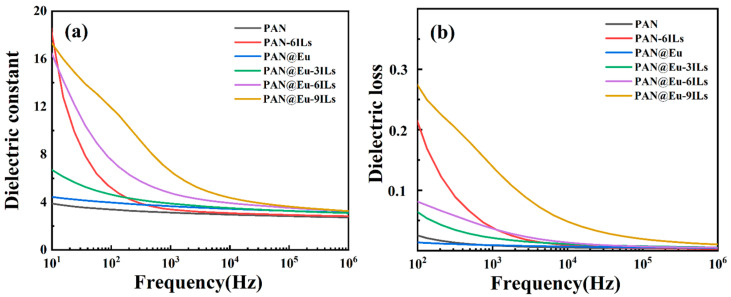
Dielectric properties of PAN@Eu-ILs composite fibers: (**a**) dielectric constant at different frequencies; (**b**) dielectric loss at different frequencies.

As shown in Figure 8a, the dielectric constant of PAN composite fibers increased significantly after the addition of ILs and Eu (NO_3_)_3_·6H_2_O. From Table 1, it can be concluded that the dielectric constant of PAN@Eu reached 3.68 at 10^3^ Hz, which was 1.5 times that of the PAN nanofibers (2.48). In addition, the dielectric constant of the dual-filler PAN composite fiber was particularly improved. When the concentration of ILs was 9%, the dielectric constant of PAN@Eu-9ILs composite fibers at 10^3^ Hz reached 6.6, which was 2.7 times that of the PAN nanofibers. The increase in the dielectric constant of PAN composite fibers in the low frequency region indicated the improvement of the interfacial polarization, which was due to the high polarity of the ILs [43,44]. In addition, the synergistic effect of the dual fillers had a positive effect on the enhanced dielectric constant. In terms of dielectric loss, the dielectric loss of PAN composite fibers increased slightly. Compared with other reported systems [45,46], this system remained at a low level (<0.14), which was beneficial for the long-term use of composite fibers as energy storage devices or piezoelectric sensors.

### 3.6. Mechanical Properties and Ferroelectric Properties

**Figure 9:**
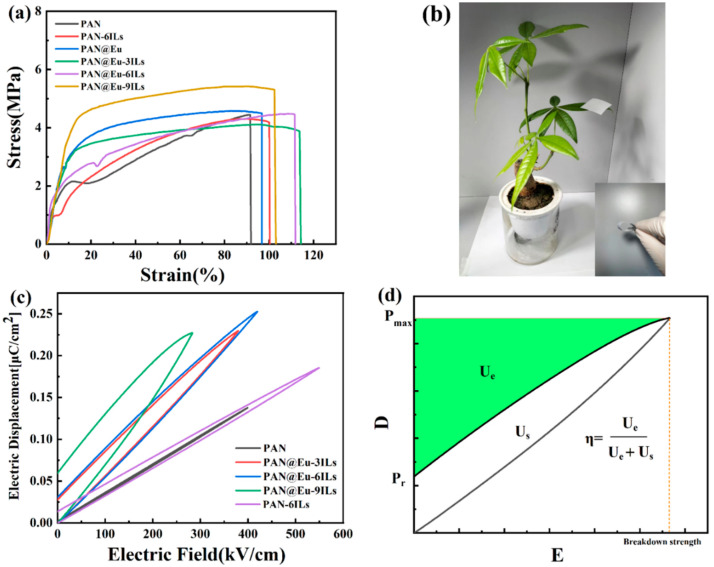
(**a**) Mechanical properties of PAN composite fibers; (**b**) DE hysteresis loop of PAN composite fiber; (**c**) photo of PAN composite fibers; (**d**) DE hysteresis loop diagram.

Figure 9a is the stress–strain curve of PAN composite fibers, and Table 1 shows the specific parameters of the dielectric properties and mechanical properties. Polyacrylonitrile nanofibers certainly exhibited mechanical strength and stretchability [47,48]. The addition of ILs made the PAN nanofibers exhibit a certain orientation under the action of electrostatic force. This had a favorable effect on the mechanical properties of composite fibers. As shown in Figure 9a, the elongation at break of PAN composite fibers was improved, and the elongation was improved more obviously by ILs. In addition, the dual fillers had a better effect on the improvement of the tensile strength and the elastic modulus of the composite fibers, and the best was PAN@Eu-3ILs, whose tensile strength and the elastic modulus were significantly lower than PAN and single-filler PAN composite fibers. The more flexible PAN composite fibers were beneficial to its application as a sensor in human health monitoring. Figure 9b shows that a PAN composite fiber with a size of 40 mm × 40 mm could easily stand on the leaf of a green plant and the leaf was not bent. In addition, the composite fiber can also withstand bending and twisting without deformation. This demonstrates the light and soft characteristics of PAN composite fibers.

Figure 9c shows the single-phase hysteresis loop of the PAN composite fiber. In the ferroelectric test, we recorded the maximum test electric field before breakdown of the sample and defined it as the breakdown strength of the sample. In Figure 9d, E is the electric field strength, D is the electrical displacement, P_max_ is the maximum polarization value, P_r_ is the remanent polarization value, U_e_ is the energy density, U_s_ is the loss energy density, and η is the energy storage efficiency. As shown in Figure 9c, the maximum polarization value (P_max_) of the dual-filler PAN@Eu-ILs composite fibers was slightly increased compared with that of the single-doped PAN-6ILs, which was the result of the synergistic effect of the dual fillers. Its effect on the breakdown strength was not obvious, but still slightly higher than that of pure PAN nanofibers. In addition, due to the influence of the dielectric loss of the PAN@Eu-ILs composite fibers, the remanent polarization value (P_r_) kept increasing with the increase in the dielectric loss, and the corresponding energy loss also increased, which seriously reduced the energy storage efficiency. When the IL concentrations were 3%, 6%, and 9%, its P_r_ values were 0.027, 0.03, and 0.06 respectively, and the energy storage efficiency values were 81%, 80%, and 60%, respectively. However, under the effect of breakdown strength and enhanced P_max_, a high energy storage density can still be maintained. Among them, PAN@Eu-6ILs had the best performance, its breakdown strength was 420 kV/cm^2^, and its P_max_ was increased from 0.14 μC/cm^2^ of PAN to 0.25 μC/cm^2^. Under this condition, its energy density (U_e_) reached 44.02 mJ/cm^3^, while the U_e_ of PAN was 26.84 mJ/cm^3^, and the energy density was increased by 1.64 times. It was obvious that the PAN@Eu-ILs system still maintained excellent energy storage performance. Compared with other reported systems [49,50], the PAN@Eu-ILs composite fibers had excellent energy conversion efficiency and good energy density. Therefore, it can be expected to be applied in the field of lightweight and high-efficiency energy devices in the future.

### 3.7. Hydrophobicity and Self-Cleaning Properties

**Figure 10:**
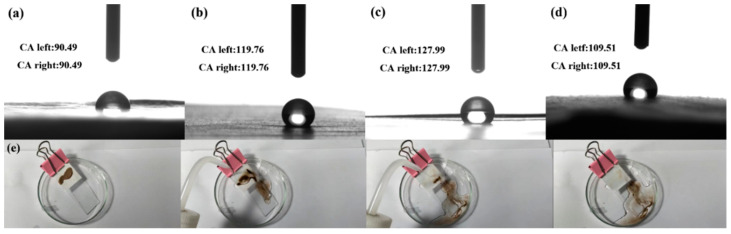
Water contact angles of (**a**) PAN; (**b**) PAN-3ILs; (**c**) PAN-6ILs; (**d**) PAN-9ILs; (**e**) self-cleaning process of PAN-ILs composite fibers.

In practical applications, the application of piezoelectric sensors may be disturbed by sweat and external moisture generated by the human body. Therefore, it was necessary to develop the hydrophobic function of composite fibers. Figure 10 shows the water contact angle of composite fibers. The water contact angle test showed that when the concentrations of IL were 0%, 3%, 6%, and 9%, the CAs of the PAN composite fibers reached 90.49°, 119.76°, 127.99°, and 109.51°, respectively. This indicated that the PAN-ILs composite fibers had excellent surface hydrophobicity. We also further explored the self-cleaning ability of PAN-ILs composite fibers. As we can see from the Figure 10e, the contaminants (sandy soil) attached to the surface of the composite fibers were quickly cleaned up under the scouring of the water flow, and the composite fiber recovered cleanly. Therefore, the PAN-ILs composite fiber had excellent performance in terms of hydrophobicity and self-cleaning. Unfortunately, the dual-filled PAN composite fiber was not hydrophobic, and it was penetrated rapidly when a droplet was dropped on the surface of the sample.

### 3.8. Fluorescence Properties and Sensitivity

**Figure 11:**
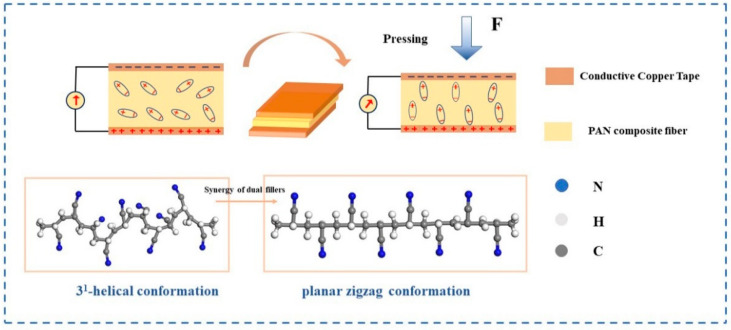
Schematic diagram of working principle of piezoelectric sensor.

Figure 11 shows the working principle model of PAN composite fibers piezoelectric sensor. It can be seen that the -CH_2_-CN- strand in the PAN was treated as a dipole, which exhibited a zigzag arrangement in the direction perpendicular to the fiber axis. In the electrospinning process, under the action of high electrostatic force, a large number of dipoles of double-filled PAN composite fibers rotated in the same direction, 3^1^ helical conformation gradually changed to planar zigzag conformation, and the internal electric potential was enhanced. At the same time, the electrons in the electrode layer exhibited opposite potential and accumulated, finally forming an external current in the wire. 

**Figure 12:**
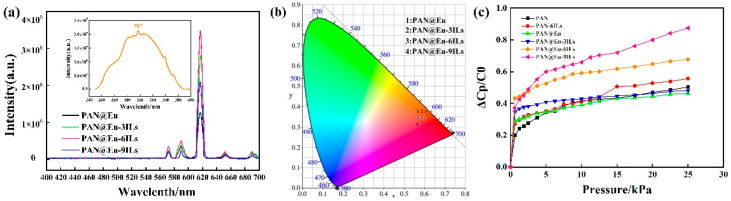
(**a**) Fluorescence properties of PAN@Eu-ILs composite fibers; (**b**) CIE diagram of PAN@Eu-ILs; (**c**) sensitivity of PAN composite fibers under different pressures.

Figure 12 shows the fluorescence spectra of PAN@Eu and PAN@Eu-ILs composite fibers. From the figure, it can be observed that all samples had diffraction peaks at 572, 590, 617, 652, and 689 nm, which correspond to the ^5^D_0_→^7^F_0_, ^5^D_0_→^7^F_1_, ^5^D_0_→^7^F_2_, ^5^D_0_→^7^F_3_, and ^5^D_0_→^7^F_4_ energy level transitions of Eu^3+^ ions. This is consistent with the fluorescence spectrum of rare earth element europium [51]. Among them, the ^5^D_0_→^7^F_2_ transition at 617 nm (red light) was the main emission peak. With the addition of ILs, the fluorescence intensity of composite fibers first increased and then decreased, and the fluorescence intensity of PAN@Eu-6ILs was the highest. However, excess ILs led to an increased probability of nonradiative transitions between Eu^3+^ ions, and in general, fluorescence properties were quenched when the probability of nonradiative transitions equaled the probability of radiative emission [52]. The corresponding band of the ^5^D_0_→^7^F_2_ transition was weakened. Therefore, the fluorescence intensity of PAN@Eu-ILs composite fibers was weakened. Figure 12b presents the CIE color coordinate diagram; the CIE color model is a color space model created by the International Illumination Commission (ILC). The point corresponding to the CIE value of PAN@Eu-6ILs nanofibers (x = 0.601, y = 0.346) exhibited red emission. The red-light properties of the PAN@Eu-6ILs composite fiber enabled it to achieve certain anti-counterfeiting and lighting functions in multi-functional sensors.

Flexible pressure sensors with simple structure and low power consumption have attracted much attention due to their promising applications in wearable electronic products. However, assembling pressure sensors with high sensitivity, low detection limit, and wide dynamic range remains to be a great challenge [53]. In order to increase the service life of the sensor device, we designed the structure of the sensor. The composite fiber was cut into a size of 2 cm × 2 cm, and the upper and lower surfaces were attached with conductive tape, and finally packaged with PET. Figure 12c shows that after adding ILs or Eu (NO_3_)_3_·6H_2_O, the capacitance change of PAN nanofibers under the same pressure was improved to different degrees. In the low-pressure region (<1 kPa), the capacitance value of composite fibers changed suddenly, and the minimum monitoring limit was less than 0.625 kPa. In the high-pressure region (5–25 kPa), with the continuous increase in the applied external force, the change in the capacitance value tended to be gentle. The sudden change in the capacitance value can be calculated by the following formula analysis (3) [54]:

(3) $$C=\varepsilon_{0}\varepsilon_{r}\frac{A}{d}$$

where *C* is the capacitance, ε_0_ is the vacuum dielectric constant, ε_r_ is the relative dielectric constant, *A* is the overlapping area between the two electrodes, and *d* is the distance between the two electrodes. It can be seen from the above formula that the capacitance of composite fibers is related to dielectric constant, contact area, and thickness [55]. The higher the dielectric constant, the greater the capacitance value. In addition, when composite fibers were subjected to pressure, their capacitance value increased with the decrease in distance. When the composite fiber was subjected to a certain pressure, the movable positive and negative charges on its surface undergo a sudden change, which was the reason for the sudden change in the capacitance value. We further analyzed and calculated the sensitivity using formula (4) [56].

(4) $$S=\frac{\sigma \left(\frac{\Delta \left(C_{P}-C_{0}\right)}{C_{0}}\right)}{\sigma P}$$

Here, S is the sensitivity of the composite fiber, *C_P_* is the capacitance value when the pressure P is applied, and *C*_0_ is the initial capacitance value. Table 2 shows the sensitivity values of PAN composite fiber. Among them, S is the sensitivity value of the low-pressure area (<0.6 kPa). It can be seen from Table 2 that the sensitivity of PAN was 0.30 kPa^−1^, and the sensitivities of PAN-6ILs and PAN@Eu were 0.43 and 0.47 kPa^−1^, respectively. In the dual-filler system, the sensitivity of PAN composite fibers first increased and then decreased with the increase in IL content. The sensitivity of PAN@Eu-6ILs reached 0.69 kPa^−1^. The piezoelectric capacitance change of the composite fibers was influenced by the synergistic effect of the dual fillers and the ILs concentration. Another explanation can be that the certain orientation of nanofibers could improve piezoelectric sensitivity [57]. However, as the pressure increased, the sensitivity decreased rapidly. For example, the sensitivity of PAN@Eu-6ILs in the high-pressure region (0.007 kPa^−1^) was much lower than that in the low-pressure region (0.69 kPa^−1^). Therefore, PAN composite fibers are suitable for piezoelectric sensors for low pressure detection, and the sensitivity of this work has obvious advantages compared with other reported systems [58,59].

## 4. Discussion

In summary, we successfully prepared an IL and (Eu (NO_3_)_3_·6H_2_O)-based PAN composite fiber. The results show that the PAN composite fibers could emit red fluorescence. Among them, the water contact angle (AC) of PAN-6ILs increased from 90.49° to 127.99° with excellent hydrophobic self-cleaning ability. Meanwhile, the PAN@Eu-6ILs of the dual-filler system exhibited good flexibility (elongation at break of 114% and elastic modulus of 2.98 MPa). In addition, the composite fibers exhibited efficient energy storage capacity. Under the action of an electric field of 420 kV/cm^3^, the energy density of PAN@Eu-6ILs was 44.02 mJ/cm^3^ and the energy storage efficiency was as high as 80%. More importantly, the composite fibers had a low detection limit (<0.625 kPa) and achieved a high sensitivity of 0.69 kPa^−1^. Overall, the multifunctional PAN composite fibers are expected to be applied in energy storage devices, optoelectronic devices, flexible pressure piezo sensors, etc.

## Figures and Tables

**Table 1:** Dielectric properties at 10^3^ Hz and specific parameters of mechanical properties of PAN@Eu-ILs composite fibers.

Sample	Dielectric Constant/10^3^ Hz	Dielectric Loss/10^3^ Hz	The Maximum Stress/MPa	Breaking Elongation/%	The Elastic Modulus/MPa
PAN	2.48	0.019	4.49	92	4.88
PAN-6ILs	4.69	0.024	4.17	100	4.17
PAN@Eu	3.68	0.030	4.48	97	4.61
PAN@Eu-3ILs	3.91	0.042	3.38	114	2.96
PAN@Eu-6ILs	4.79	0.045	4.47	112	3.99
PAN@Eu-9ILs	6.60	0.139	5.35	102	5.25

**Table 2:** Sensitivity values of PAN composite fibers under the action of low pressure and high pressure.

Sample	S/kPa^−1^
PAN	0.30
PAN-6ILs	0.43
PAN@Eu	0.47
PAN@Eu3ILs	0.56
PAN@Eu-6ILs	0.69
PAN@Eu-9ILs	0.60

## Data Availability

Not applicable.
